# Opening Lecture at the Twenty Third Annual Course of Lectures of Rush Medical College

**Published:** 1865-11

**Authors:** J. V. Z. Blaney

**Affiliations:** Professor of Chemistry and Pharmacy


CHICAGO
MEDICAL JOURNAL.

VOL. XXII.] NOVEMBER, 1865. [No. 11.

### ORIGINAL COMMUNICATIONS.

### OPENING LECTURE
AT THE
TWENTY-THIRD ANNUAL COURSE OF LECTURES
RUSH MEDICAL COLLEGE.

By J. V. Z. BLANEY, M. D.,

*Professor of Chemistry and Pharmacy.*

[Published by request of the class.]

GENTLEMEN:—

I have the honor to have been selected by my colleagues to convey to you their cordial welcome to the Lecture Rooms of this Institution, and to express their satisfaction that so many who have previously, and so many who have not heretofore, attended their lectures are present, to exchange greetings with them, on this the first day of the Twenty-third Annual Course of Lectures of Rush Medical College.

It is with much pleasure that, after an absence of several years, I find myself once more restored to peaceful avocations and resuming duties which, for years past, I have performed in this institution. Though I have regretted the long absence which I thought was imposed upon me by my duty to the Federal Government, after that I had once enrolled myself on the Medical staff of the army of the Union, I can not regret for a moment the experience which I have had, or the humble service which I have been privileged to render in the glorious cause of the assertion of the integrity of the Union of these United States, and the establishment of universal freedom to all those who respect the rights of other citizens of a common country.

Gentlemen, our country has passed successfully through a crisis and a contest such as no other country, of ancient or of modern days, has ever experienced. Those only, who have taken part in the contest, can appreciate the fierce fury of the conflict when Greek met Greek. At last victory has perched upon our banners, and peace with her genial influences, has once more kindly resumed her sway over our great and still glorious republic. The fierce onslaught of contending hosts has passed; the roar of artillery and the rattle of musketry is no longer heard in the land; the bulletins no longer report the thousands of killed and wounded. Thanks be to God, the struggle of brethren who should have been linked in the most indissoluble fraternal bonds, for the common good, and for the cultivation of peaceful arts, is over, and a sweet, gentle peace falls, like a soft and kindly twilight, over all the land, hushing to silence all discordant sounds, and preparing all for the sweet repose which follows unusual exertion. God grant that the dawn of a new era, when science, literature, and art may flourish as never before, may be the result of the bright prognostics of the sunset of our late lamentable, but unavoidable struggle.

Gentlemen, those of you who are about to prepare yourselves for a professional life, if living to the ordinary age usually granted to those who pursue the liberal professions, will see great things. Your lines have fallen in pleasant places. Do your duty, and your whole duty to yourselves in preparation for your work, and the times themselves will afford you opportunities for success, for usefulness, and for personal celebrity, such as your predecessors in the Medical profession have never known. Only be true to yourselves and community will recognize and appreciate your qualifications, and demand your services.

Your opportunities for acquirement of the principles of the profession you have chosen are almost unlimited. All the knowledge which has been accumulated by ages of observation and experience, is at your command. It has been quaintly said that children begin where their parents leave off. If or not this be true in the domestic relations, it is eminently true when predicated of the liberal professions, provided always that the pupil avails himself, to the extent of his ability, of the experience and knowledge acquired by his predecessors. Excepting under oppressive governments, which have temporarily stifled its efforts, science has never gone backward. Only in the dark ages were the arts lost. In a liberal age, and in a liberal community, under a liberal government, science always advances. Every age adds to her treasures, and upon those who have the privilege of benefiting by the labors of a previous age, rests the responsibility, not to themselves alone but to the age in which they live, to community, to science herself, of whom they profess to be votaries, that they will not squander, but will add to the bountiful wealth which has been prepared for their entertainment; a wealth of knowledge earned by the labors of thousands, who have worked earnestly and faithfully in her cause, many of whom have gone to their last rest, unknown and unsung, but will reap the ripe fruits of their labors in the cause of humanity in a better and more appreciative atmosphere.

Gentlemen, I do not propose to attempt to impress upon you the importance to our common humanity of the studies in which you are about to engage, or the necessity of rendering yourselves proficient as far as practicable, before engaging in the actual practice of your chosen profession. Your presence here with the intention severally to devote your lives to the practice of the Medical profession, sufficiently attests your appreciation of the importance of proficiency, and the value to community of the science which you have espoused. It would, then, be almost an insult to you to present on this occasion the benefits which our noble profession has heretofore, and still continues to confer upon mankind, though I regret to say that frequently the individuals benefited by their labors are so unappreciative as not to recognize the man of scientific acquirements, profound investigation and solid attainment, from the miserable empiric whose foul advertisements taint the atmosphere and corrupt the moral sense of intelligent communities. But so it must ever be, so long as vice and immorality exist in our large cities and superficiality in the intellectual training of our youth, so long also as wealth is to be considered the first object to be obtained, and so long as vice is winked at and even apologized for in public society.

True science can only flourish and receive its proper appreciation when society is itself intelligent, and its morals pure. In the foul and pestilential atmosphere of a polluted and crowded population, all kinds of vermin and parasites find the proper conditions for their developement, existence and reproduction, and all precautions which may be taken to prevent contagion will fail, unless the atmosphere is purified and the conditions changed. Hence it is that Quackery, from the most infinitesimal to the most magnificent of its various forms, finds its home in large cities, where there exists the most perverted ideas of morals and the grossest exhibitions of vice. Under such conditions it is curious to observe that the public press, which professes to be, and should be, the censor of the morals of community, lends itself to the diffusion and perpetuation of these swindles on the masses. But still more to be reprobated and regretted, members of other liberal professions, (there are, I am happy to say, most honorable exceptions,) ignorantly, but no less injuriously, lend themselves by certificates to quack advertisements and recommendations of nostrums, to perpetuate and encourage the inherent tendency of the masses to accept empirical systems and employ empirical practitioners. But enough of this, the foe, if foe it be, is not worthy of our fire, except to protect the ignorant. Our ammunition is wasted, if we contend with these excrescences on society. Guerillas should be hung, not shot. So long as there are fools there will be quacks; the millenium is not yet.

Now, if your desire is to be and become learned and honorable members of the medical profession, a course of study is to be pursued which should be well laid out, your plans drawn and your determination fixed to follow that plan to its consummation. And in this regard, I have first to say that a preliminary education is of the first and highest importance to every student, not only of our own but of any liberal profession. As regards the amount of previous education required to enable the student to master the profession of medicine, authorities and the committees of conventions and associations differ, but all agree that a good grounding in all the branches of an ordinary English education is absolutely necessary to success, and that some knowledge of the Ancient Latin and Greek languages, materially facilitate the acquisition of the principles of our profession. Excepting in unusual instances where genius has overridden the rules which govern the general proposition; the cases are rare in which high position has been attained without preliminary education.

Permit me then, gentlemen, to exhort you that, if you feel the want of previous education, you will endeavor, without delay to repair your deficiency; with industry, it may be done while still pursuing your course of studies, delaying somewhat, perhaps, your reception of your diploma, but, if effected, on entering the ranks of the profession at the completion of your course of studies, it will be with advantages which tenfold repay you for the delay. I have been incited to make these remarks because of the fact that there is a disposition, stronger perhaps, in the West than in the older and more developed portions of our country, an almost unconquerable disposition, in young men especially, to push forward to the goal they have set before them, and that with insufficient training before entering the arena. In the Olympic games of ancient Greece severe training was submitted to before the contest was accepted, or the contestants permitted to enter the course. This haste, except in extraordinary cases where genius surmounts all precedents, is not only injudicious, but almost invariably condemns a man, whatever his industry, to simple mediocrity for a long period of his early professional life, and unless he has a powerful will, strong hope and unflagging industry, he is apt to despair of success, while inferior minds, with the advantage of a good preliminary education, will distance him in the race.

The most important step in your advance to professional attainments is this: That you become thoroughly grounded in the rudimentary branches of the profession. First in order of importance, perhaps, comes Anatomy — the science which teaches the structure of the human frame, that most perfect of all machines which God himself has yet constructed, wonderful in the beauty of its proportions; wonderful in its adaptation to the varied uses demanded of its several parts; wonderful in its power of self-repair; wonderful in its resistance against abuse of natural laws; wonderful that it can, in exceptional cases, exist a hundred years, resisting all diseases, all climates, and all conditions of life; still more wonderful in its connection with and dependence upon that invisible essence, the soul of man, which controls, modifies, regulates, or abuses this most wonderful of all machines.

Should the superintendent of any of our railroad companies employ, to take charge of and manage a locomotive engine, a man who was not fully and perfectly conversant with all its parts, the object of each and every part, the mode and manner in which the vital energy of heat converting water into steam acts upon and through each valve, on every lever, on every crank, and on every wheel, he would be thought derelict of duty, and held responsible by an indignant community, for the loss of life and property consequent upon the ignorance of his employee. Yet, forsooth, any ignoramus who, by flaming advertisements, and fulsome self-adulation, obtrudes himself upon public notice, will find fools who will trust that most perfect, most intricate and delicate piece of workmanship to the hands of a man, or rather a madman, who never saw the interior of the machine, has no knowledge of its mode of working, does not know where are its valves, or what the power, or how it propels its separate parts—in a word, is as totally ignorant as a child unborn of the subject he is acting on, or the means he is using to remedy its aberrations,

Suppose, gentlemen, that either of you had a valuable watch, perhaps an heirloom in your family. It has become deranged in its movements; it is not reliable in recording the hour; what would you do? Would you go to a blacksmith to have it repaired? or to a scissors-grinder? No, you would say—this man may be expert to shoe my horse, but he may not touch my watch. And why? Because he does not understand its anatomy and physiology. You would even hesitate to employ a watch-makeor until you were certified by indisputable references that he understood thoroughly its mechanism, and how to remedy the derangement of your treasure.

Yet, strange as it may seem, there still exists in this nineteenth century, men who profess to practice upon the intricate mechanism of the human body, who have never seen and who have not the slightest knowledge of its interior structure. Truly it has been said that it is strange that "a harp of a thousand strings should stay in tune so long."

The science of Anatomy stands, as I have said, at the foundation of medical science. It is the corner-stone, and upon its faithful acquirement depends the whole success of the future candidate for professional honors. Having become proficient in this branch of the profession, the department of Physiology next claims your attention. It will teach you the mode of operation of the vital forces, which act upon and keep in constant motion this intricate machine.

The study of Pathology will instruct you in the aberrations in the movements, and the changes of structure which result from disease. Therapeutics, Materia Medica and Chemistry, will supply you with a knowledge of the mode of action, and the materials with which to remedy such aberrations. Surgery supplies and directs the application of the instruments in the treatment of those cases and conditions in which the more subtle remedies of the Pharmacopœia fail to relieve, which for the most part are injuries which occur from the applications of external force, or from hidden causes which produce abnormal growths and excrescenses. Obstetrics will inform you of the laws of birth and generation—the starting point of human existence—and the management of delivery, or the ushering of this fleeting body into this fleeting world.

The practice of Medicine is the department which instructs you how to diagnose disease, to recognize the existence of various pathological changes, and suggests the remedies to be applied, which are furnished by the Pharmaceutist. With all and each of these branches must you become familiar before you can enter even the threshold of the Medical Profession.

Now, gentlemen, with this curriculum laid out before you, you must perceive that the term allowed for the existence of any individual is too short to permit the acquisition of a perfect knowledge of every branch of this vast field of study. In the older and more developed countries of Europe, the subdivision of labor and of studies has come to be recognized as the main condition essential to success. In this country, where society may be said to be yet in a transition state from the more primitive to the most cultivated, the subdivision of studies, so far as the liberal professions are concerned is yet looked upon with jealous eyes, and the man who dares to announce that he has devoted his time, his labor, and his life, to the special cultivation of a particular department of Medical Science, is by many, not only of the uninformed, but by men of standing in the profession itself, characterized as approaching, if not actually having arrived at, the position o an Empiric. Never was there a greater mistake. There is an ancient proverb which says, "Beware of the man of one book," which strictly applies to the matter of specialities in the medical profession.

If it be true that no one can perfect himself in all, why should he not in one branch of study? Instead of a feeble incompetency, he could offer himself as a competent servitor to the public in the matter of his specialty. For one I advise that you select for yourselves, as soon in your course as practicable, a particular branch of medical study to which, after the due acquirement of the rudimentary branches, you propose to devote yourself. Having made your selection, let your course of studies thereafter, while not neglecting others, tend specially to that branch which you may have selected as your speciality. You will thus probably, if you have ordinary ability, attain eminence when otherwise you would be obliged, by diluting your efforts in the attempt to turn your attention into too many channels, fall into a meagre mediocrity.

I have endeavored, gentlemen, to give you a simple outline of the course of studies you are about to adopt, and briefly to indicete to you your duty to yourself and the profession.

Take as your maxim the ancient aphorism, "*Ars longa vita brevis est*"—Art is long and life is fleeting. Remember that no one mind can comprehend or bring into useful application all of science. That in the study of Medicine you have, as were, three separate problems to solve—Life, Disease, Death. Your object is to preserve Life, to prevent or cure Disease, and to prevent death. This is the task, and these the problems you have to solve as Students of the Medical Profession. Are you prepared, and will you submit to the labor necessary to render yourselves competent to perform these duties? are the questions that I leave for your own consideration.

